# Response to “understanding the behavioural determinants of opioid prescribing among family physicians: a qualitative study”

**DOI:** 10.1186/s12875-020-01187-w

**Published:** 2020-06-13

**Authors:** Jason W. Busse, David Juurlink, D. Norman Buckley, Gordon H. Guyatt

**Affiliations:** 1 Department of Anesthesia, Michael G. DeGroote School of Medicine, McMaster University, HSC-2V9, 1280 Main St. West, Hamilton, ON L8S 4K1 Canada; 2 Sunnybrook Health Sciences Centre, 2075 Bayview Ave., Room G1 57, Toronto, ON M4N 3M5 Canada; 3 Department of Anesthesia, Michael G. DeGroote School of Medicine, McMaster University, MDCL-2104, 1280 Main St. West, Hamilton, ON L8S 4K1 Canada

## Abstract

The 2017 Canadian opioid Guideline made both strong recommendations, indicating that all or almost all fully informed patients would choose the recommended course of action, and weak recommendations, in which different choices are appropriate for individual patients based on their values and preferences. The Guideline’s recommendation to taper legacy patients prescribed high-dose opioid therapy is weak, and mandatory tapering is expressly discouraged.

## Main text

We read with interest Dr. Desveaux and colleagues’ qualitative study regarding barriers to, and enablers of, guideline-concordant opioid prescribing for chronic non-cancer pain [1], and appreciate the opportunity to comment.

The authors’ description of the 2017 Canadian Guideline’s recommendations does not make the distinction between weak and strong recommendations in the Guideline, except to note the Guideline “strongly suggest[s] that the maximum prescribed dose be restricted to less than 50 mg of morphine equivalent dose when opioid therapy is being initiated”. Recommendation 7 (which recommends against prescribing ≥50 mg morphine equivalent per day for patients beginning opioid therapy) is classified as a weak recommendation, meaning that the majority of informed patients would choose the recommended course of action, but an appreciable minority would not. With weak recommendations, clinicians should recognize that different choices will be appropriate for individual patients, and they should help patients arrive at decisions consistent with their values and preferences and clinical needs.

The authors assert that the Guideline recommends “… restricting the maximum prescribed dose to 90 mg morphine equivalents daily.” Critically, however, this guidance (Recommendation 6) applies only to patients commencing opioid therapy and does not apply to legacy patients who have already escalated to higher doses. Moreover, the remark associated with this recommendation in the Guideline states: “Some patients may gain important benefit at a dose of more than 90mg morphine equivalents daily. Referral to a colleague for a second opinion regarding the possibility of increasing the dose to more than 90mg morphine equivalents daily may therefore be warranted in some individuals” [2].

Some of the physicians interviewed for the authors’ study expressed frustration that many of the Guideline’s recommendations - 6 of 10 - were ‘weak’ [2]. Clinicians might indeed prefer unequivocal direction, but there are many situations in which decisions are value and preference dependent and the optimal course of action differs across patients; typically, because of low certainty in the evidence for a critical outcome, or because the benefits and harms are closely balanced. In such situations, unequivocal recommendations that fail to mandate shared decision-making (as weak recommendations do) would lead to suboptimal patient care. Forcing reductions in opioid dosing in legacy patients experiencing pain control with minimal side effects and aware of the arduous process of reduction, sometimes fraught with risk, and thus very reluctant to reduce dosage, provides one example.

Some physicians in the study raised concerns about the lack of recommendation for screening tools to inform the decision whether or not to prescribe opioids, which the 2010 Canadian Guideline contained [3]. We made no such recommendations, because there is no evidence supporting the validity of opioid prescribing screening tools - a finding reinforced by a 2019 systematic review [4].

Some physicians felt that Recommendation #10 (strong recommendation for a formal multidisciplinary program for patients with chronic noncancer pain who are using opioids and experiencing serious challenges in tapering) is impractical. We recognize that this recommendation is resource-dependent, which is why the Guideline highlighted an alternative of a coordinated multidisciplinary collaboration including health professionals whom physicians had access in their community.

Some physicians felt that following the 2017 Canadian Guideline “…encouraged actions that led to destabilization of otherwise stable patients…including the use of illicit drugs in response to opioid tapering.” We share these concerns, and have highlighted this issue as it relates to overly aggressive adoption of the 2016 Centers for Disease Control and Prevention Guideline for Prescribing Opioids for Chronic Pain [5–7]. The 2017 Canadian Guideline explicitly discourages inappropriate opioid tapering, a position informed by our guideline’s values and preferences statement [8] – note the example of the weak recommendation above. If followed, the 2017 Canadian Guideline will not result in forced opioid tapering or severe opioid withdrawal that may lead patients to seek relief from other dangerous sources.

The Canadian Guideline is available here in an interactive, multi-layered format, with patient decision aids for all weak recommendations: https://www.magicapp.org/public/guideline/8nyb0E

We reiterate our view that, if followed, the 2017 Canadian Guideline will promote evidence-based prescribing of opioids for chronic non-cancer pain.

## Data Availability

Not applicable.
